# Supplementary figures and images for: Structural basis of Blastomyces Endoglucanase-2 adjuvancy in anti-fungal and -viral immunity
Source: PLoS Pathog. 2021 Mar 18;17(3):e1009324. doi: 10.1371/journal.ppat.1009324 (PMC8009368; doi:10.1371/journal.ppat.1009324)

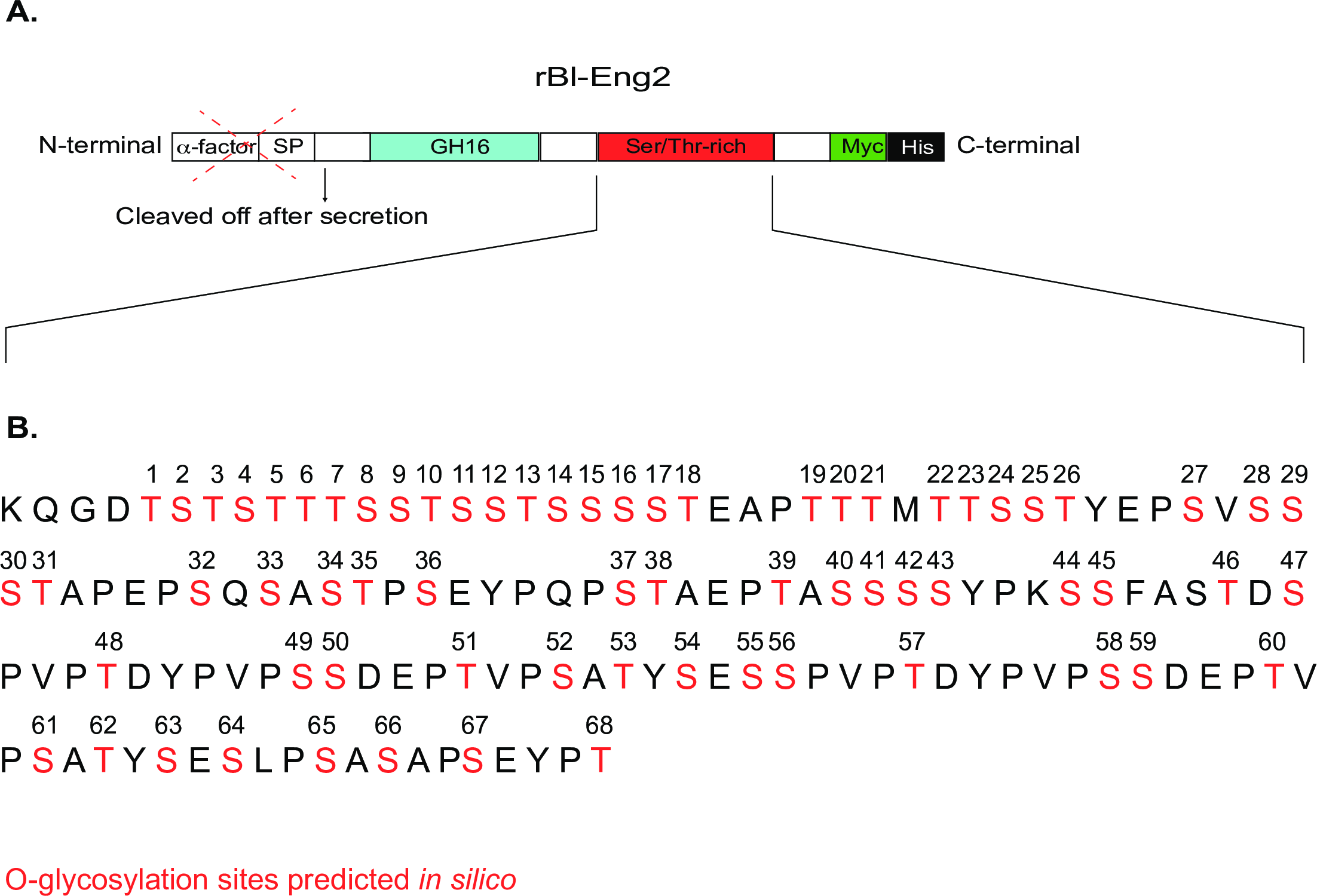

Supplement: S1 Fig — A) Schematic of rBl-Eng2 domain structure. The protein consists of the catalytic glycosyl hydrolases family 16 domain (GH16, blue) and a Serine/Threonine-rich region (red). The alpha factor signal promotes expression in Pichia and the Myc tag (red) and 6x Histidine tag (black) enable purification. (B) Amino acid sequence of the Ser/Thr-rich region predicts the presence of 68 O-glycosylation sites (in red and numbered). (TIF) [file ppat.1009324.s001.tif]

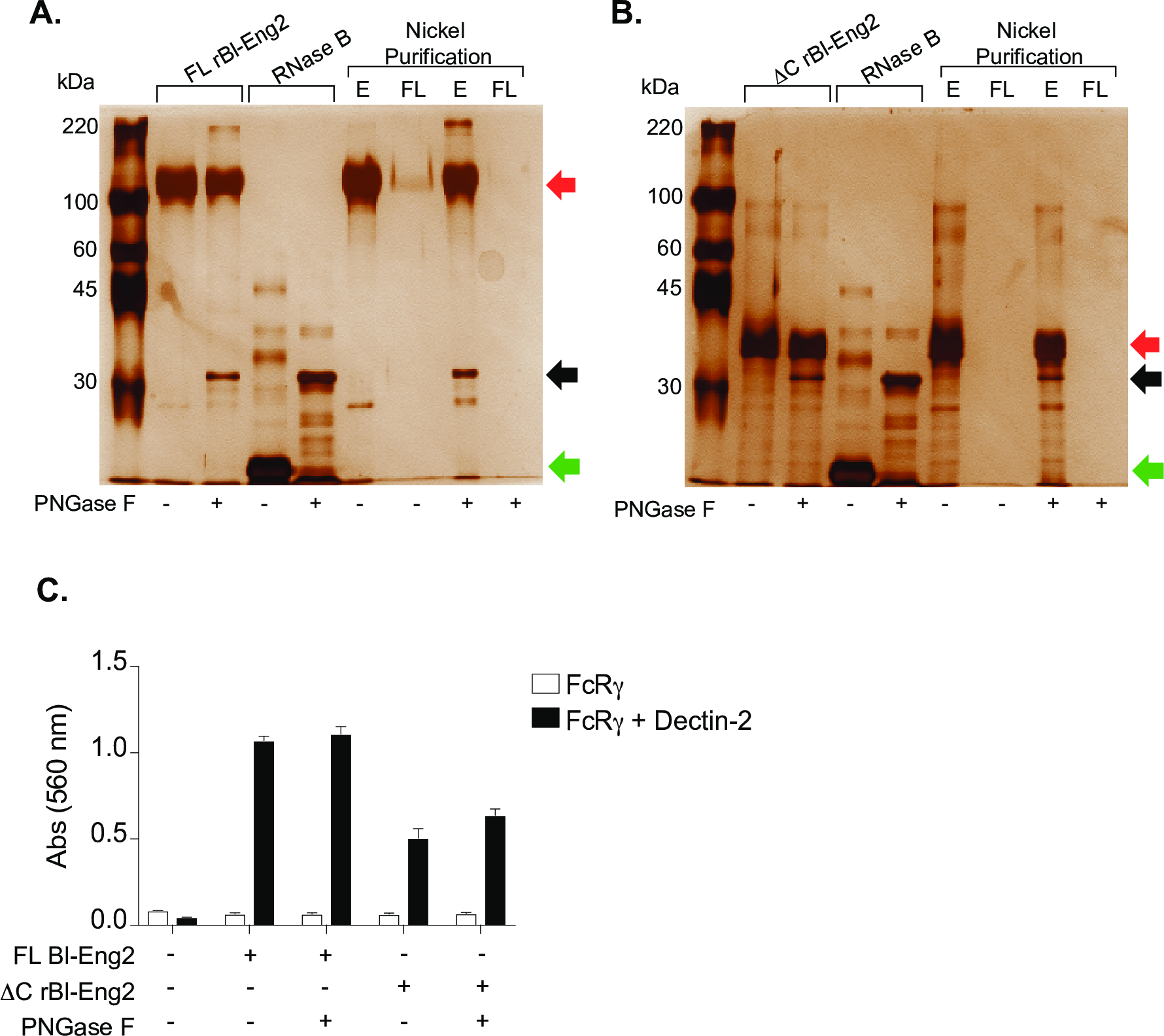

Supplement: S2 Fig — SDS-PAGE of nickel purified, full-length rBl-Eng2 (A) and ΔC rBl-Eng2 (B) treated with PNGase F. The gel was stained with silver nitrate. RNase B was used as control to demonstrate that PNGase F was functional. Red arrows = rBl-Eng2. Black arrows = PNGase F. Green arrows = Rnase B. E = eluate. FL = flow-through. C) Purified proteins (E, eluate) were tested in the Dectin-2 reporter assay. The data are representative of three independent experiments. (TIF) [file ppat.1009324.s002.tif]

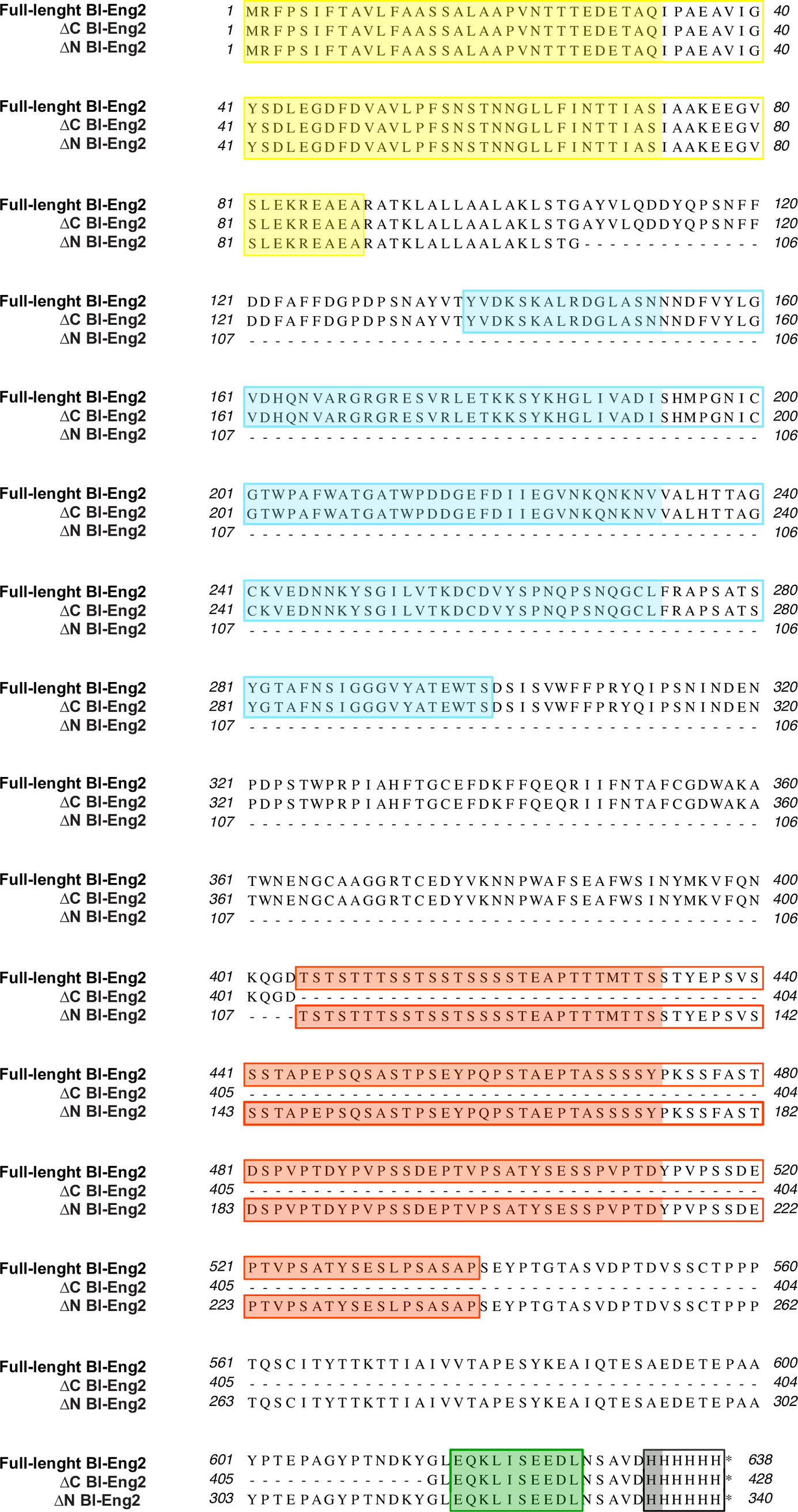

Supplement: S3 Fig — The amino acid sequences of the full length, ΔC and ΔN Bl-Eng2 fusion proteins expressed in Pichia are shown aligned to illustrate the deleted residues (-). The initiator methione of the native Bl-Eng2 protein has been removed in these constructs. Domains of interest are highlighted in color boxes. Yellow, Saccharomyces cerevisiae alpha factor secretory signal peptide sequence; cyan, GH16 domain of Bl-Eng2; red, serine/threonine-rich domain of Bl-Eng2; green, c-Myc epitope tag; black, poly-histidine tag for purification; *, stop codon. (TIF) [file ppat.1009324.s003.tif]

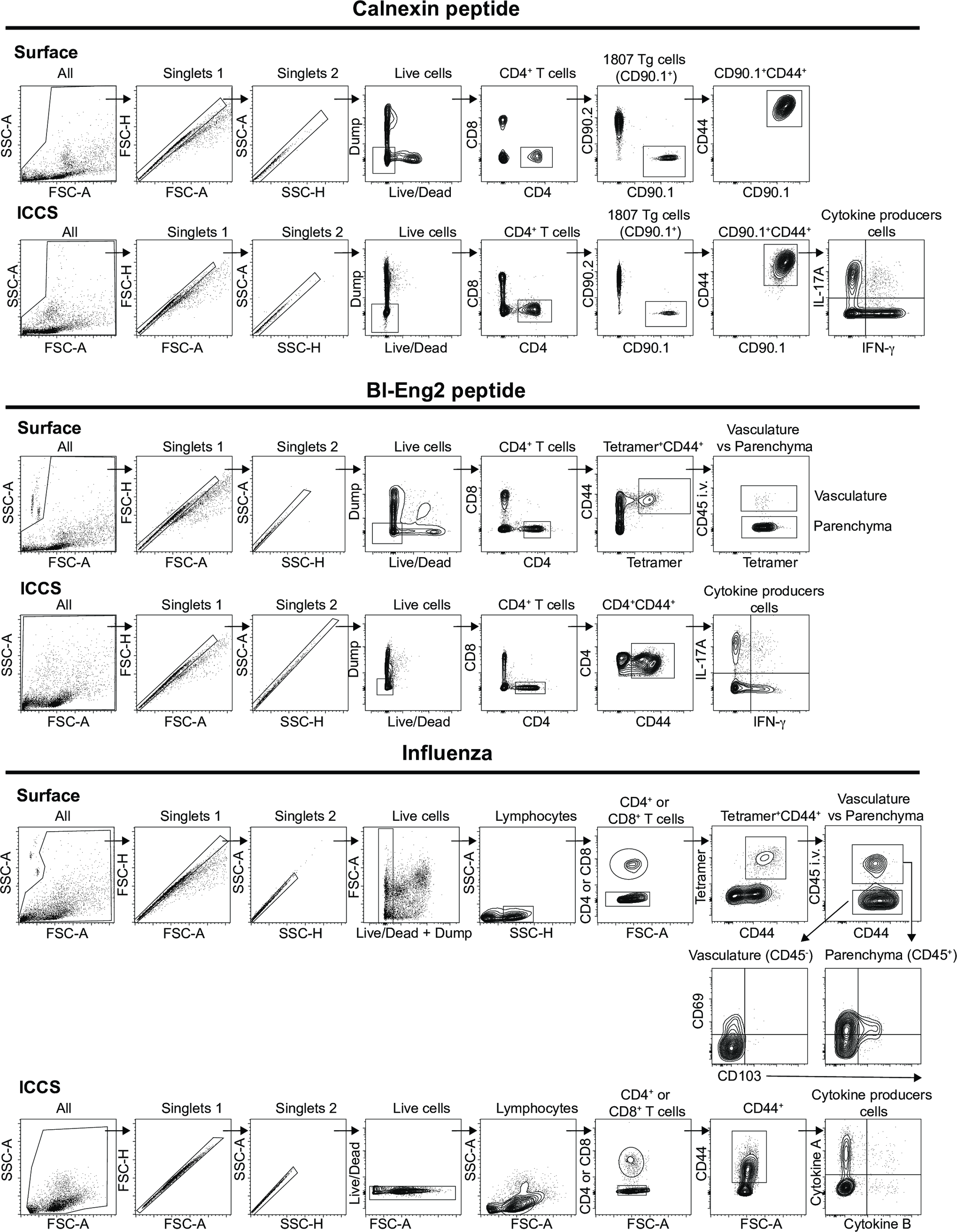

Supplement: S4 Fig — The gating strategy for calnexin- (top), Bl-Eng2- (middle) and NP (bottom) -specific T cells was as follows: FSC and SSC gate was used to eliminate counting beads, singlets were used to eliminate duplets and aggregates; a dump channel composed by CD11c, CD11b, NK1.1 and B220 was used to exclude myeloid cells, NK cells and B lymphocytes; a live stain eliminated dead cells; live cells were then separated into CD4+ and CD8+ T cells. CD45 mAb was injected intravenously 5 minutes before euthanasia to mark cells in the vasculature. (TIF) [file ppat.1009324.s004.tif]

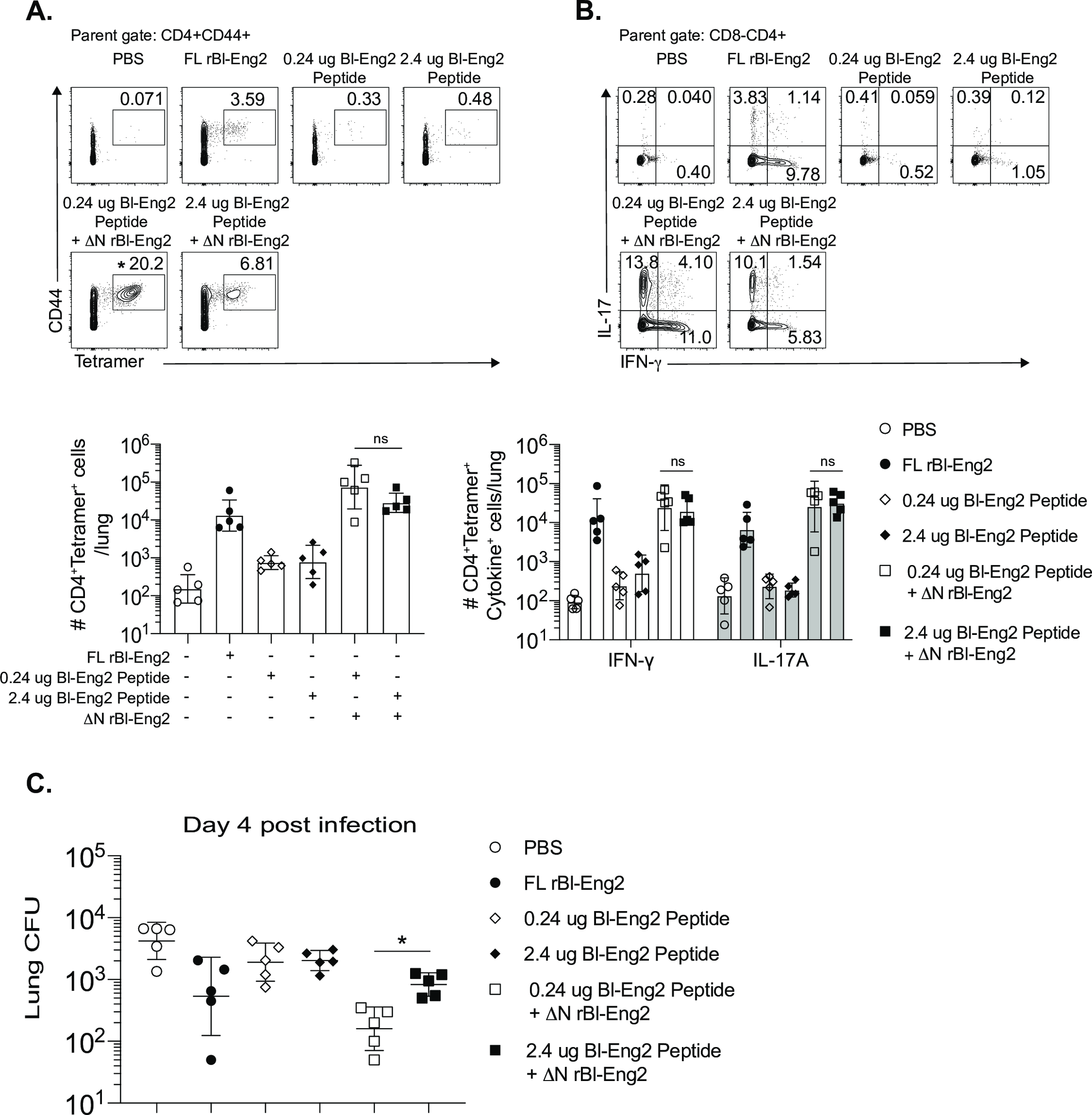

Supplement: S5 Fig — Mice were subcutaneously vaccinated with 10μg ΔN rBl-Eng2 (as adjuvant) emulsified in IFA and with equimolar (0.24 μg) or 10 times more peptide (2.4 μg) relative to the 10 μg full-length rBl-Eng2 (used as control). Two weeks after the vaccine boost, mice were challenged with B. dermatitidis. At day 4 post-infection, the frequency and number of total tetramer+ T cells in the lungs (A), and IL-17 and IFN-γ producing cells (B) were analyzed by flow cytometry. Data shown are from one representative experiment of two performed (n = 5 mice/group). *p<0.5 vs 2.4 μg Bl-Eng2 peptide. C) Lung CFU are displayed as the geometric mean with standard deviation. Data are from one representative experiment of two performed (n = 5 mice/group). *p<0.05 vs. 2.4 μg Bl-Eng2 peptide. (TIF) [file ppat.1009324.s005.tif]

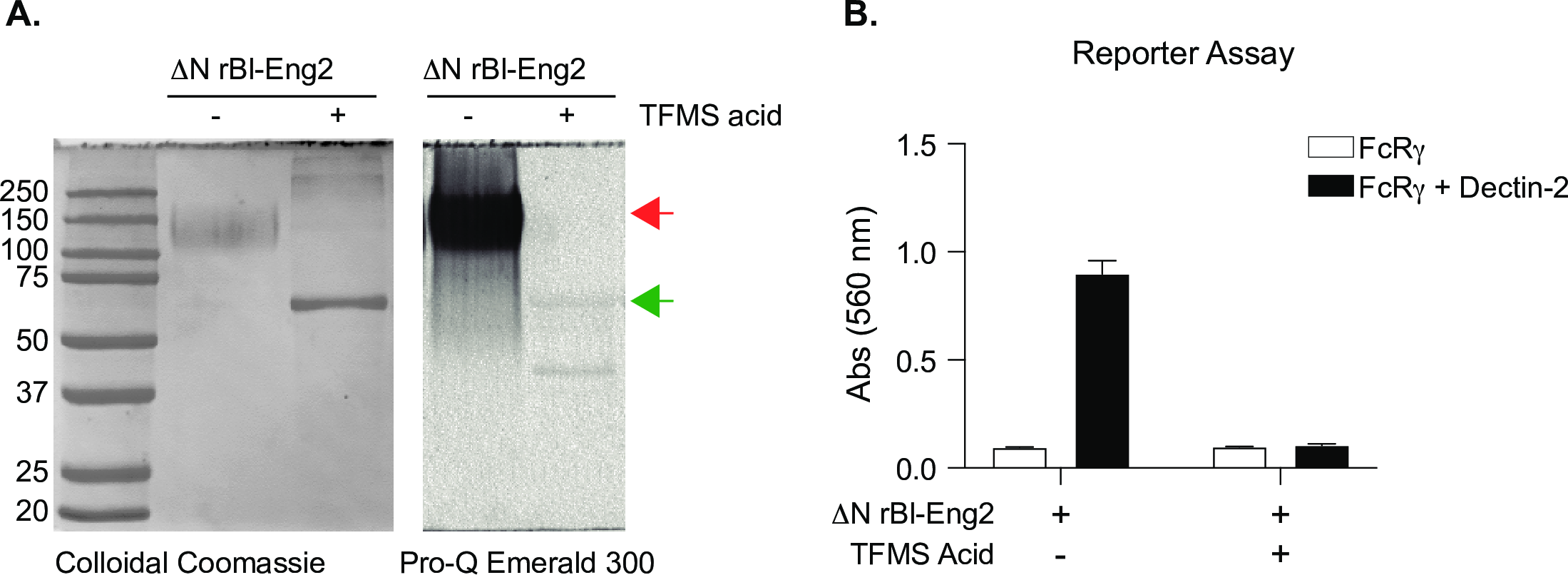

Supplement: S6 Fig — A) SDS-PAGE of ΔN rBl-Eng2 before (red arrow) and after (green arrow) deglycosylation with trifluoromethanesulfonic acid (TFMS acid). The gel was stained with colloidal Coomassie (blue) and Pro-Q Emerald 300 (gray) to evaluate the molecular weight and the presence of sugar, respectively. B) Dectin-2 reporter assay of deglycosylated ΔN rBl-Eng2. (TIF) [file ppat.1009324.s006.tif]
