# Supplementary material for: Structural basis of Blastomyces Endoglucanase-2 adjuvancy in anti-fungal and -viral immunity
Source: PLoS Pathog. 2021 Mar 18;17(3):e1009324. doi: 10.1371/journal.ppat.1009324 (PMC8009368; doi:10.1371/journal.ppat.1009324)
Supplement: Attachment — Submitted filename: Rebuttal letter 2021 final.docx [file ppat.1009324.s007.docx]

January 12, 2021

**RE: Resubmission of manuscript D-20-01377**: " Structural basis of *Blastomyces* Endoglucanase-2 adjuvancy in anti-fungal and -viral immunity"

Dear Editors:

We are pleased that the reviewers agreed on the careful nature of the study and quality of the presented data. Thank you for providing us additional time to respond fully and with new data to the concerns that were raised. We completed the requested new experiments and revised the paper in response to the reviewer’s concerns and your request.

***Editorial comments:*** *A novel finding relates to the identification of distinct segments of Blastomyces endoglucanase that contain separable T cell-stimulating and adjuvant properties. However, there were differences in opinion regarding the novelty of the work and its physiologic relevance in vaccine immunity beyond the Blastomyces infection model. I believe that is important for the authors to respond to points raised by reviewer 2, including a virologic readouts in the influenza model. The title should be re-worked to reflect better the findings of the study - at this time, the authors do not have compelling data for an effect on anti-viral immunity outside of stimulating T cell responses.*

**Response:** We summarize the new experiments and data just below for your and the reviewer’s convenience. We also offer a point-by-point response to the questions raised by the reviewers. We indicate all changes to the paper by highlighting modifications in yellow in a marked copy of the revised manuscript.

**Summary of new experiments performed and fresh data added to the paper**

- **Two new models of fungal infection**: Studies to investigate whether ΔN Bl-Eng2 harbors adjuvant activity against other fungal pathogens beyond the *Blastomyces* infection model (new Figure 6). We added new models of systemic candidiasis and pulmonary cryptococcosis.
- **Evidence for anti-viral resistance:** Analysis of the viral burden following vaccination with NP antigen, Adjuplex and ΔN Bl-Eng2 and challenge with Influenza (new Figure 7).
- **Studies to investigate the role of IFA** in formulating antigen and ΔN Bl-Eng2 for vaccination (see Fig. 1 below in the rebuttal letter).

**REVIEWER #1:**

This reviewer had no major comments, but did raise minor comments:

***Query 1:*** *References 23 and 27 are the same.*

**Response:** The reviewer is correct. We eliminated the duplicated reference.

***Query 2:*** *In the introduction, where the authors talk about vaccine-induced T cell immunity against fungi, they only refer to their own work on dimorphic fungi (which is undoubtedly of great relevance), but they should also include work from other fungi, e.g. C. albicans Als3.*

**Response:** We corrected the oversight and now cite several articles on Als3 induced vaccine immunity to infection with *C. albicans* and *C. auris* in the Introduction.

***Query 3:*** *Figure 1A shows >2 bands for the E. coli-expressed rBl-Eng2, without the authors commenting on it.*

**Response:** The reviewer is correct. Fig. 1A shows a double band of *E. coli* expressed recombinant Bl-Eng2 protein after purification over a nickel agarose column. The expression construct in *E. coli* is a fusion protein comprised of Bl-Eng2 and NusA, which has been used to facilitate secretion of the fusion protein. For purification of the fusion protein, we placed a histidine and c-Myc tag at the C terminus. In order to attempt to get a single band after purification, we eluted the protein with different concentrations of imidazole and adding imidazole to the binding buffer. However, we could not get rid of the lower molecular 70 kDa band. We have not tried to identify the lower molecular band, but it is conceivable that it is NusA since the expected Mr=66 kDa. The higher molecular weight band is the fusion protein containing Bl-Eng2 based on western blot analysis. Most importantly, a titration of *E. coli* expressed Bl-Eng2 (non-glycosylated), using up to 240 ng/well, did not stimulate the reporter cells, whereas 30 ng/well of glycosylated Bl-Eng2 from Pichia triggered Dectin-2 signaling. We now state in the revised manuscript, that the higher molecular band (boxed in red) in Fig. 1A contains recombinant Bl-Eng2 (please see page 20 of the manuscript).

***Query 4:*** *Figure 6: In some figure panels, certain symbols look like they could be outlayers (e.g. Fig 6B, NP366 and NP311-specific events in the NP + DeltaN-rBl-Eng2 + Adjuplex-treated group (Parenchyma); Fig. 6DNP45 47 52-specific IFNg production in the NP + DeltaN-rBl-Eng2 + Adjuplex-treated group). Did they authors perform an outlayer analysis? In case, outlayers are found, they should be excluded and statistical significances re-calculated.*

**Response:** In response to reviewer 2 and the editors, we repeated the vaccination with ΔN rBl-Eng2 in the Inluenza model to include resistance data. We are now showing the results of two independent experiments (with female and male mice) in Fig. 6. We did subject the new data to an outlier analysis using the ROUT method with 1, 2 or 5% of aggressivity. We did not find any outliers.

***Query 5:*** *Supplementary Figure 5: Authors show the statistical significance (resp. the non-significance) for the difference between the last to groups. What about the difference between FL rBl-Eng2 versus the combination of the BL-Eng2 peptide + DeltaN-rBL-Eng2 (which is at the centre of what this experiment addresses)?*

**Response:** The main purpose of the supplementary figure 5 (now SFig. 4 in the revised manuscript) is to provide a justification for the amount of peptide we used to vaccinate mice in Figs. 4+5. We vaccinated mice with full length Bl-Eng2 for two reasons: 1) as a positive control since we have previously demonstrated that the full-length protein harbors adjuvant activity, and 2) we based the amount of peptide used for vaccination on equimolar amounts of peptide present in the full-length protein (0.24 μg). Since we didn’t know whether peptide will be subject to increased degradation *in vivo*, we vaccinated an additional group of mice with 10 times as much peptide (2.4 μg). The data in SFig. 4 indicate that the lower (equimolar) amount of peptide was equal or better in facilitating priming of Th1/17 cells and reducing lung CFU than the higher amount of peptide, when combined with ΔN rBl-Eng2 adjuvant. Consequently, we statistically analyzed whether vaccination with the two amounts of peptide was different. It turned out that the lower amount of peptide was equally suited to prime tetramer+ Th1 and Th17 cells and better in reducing lung CFU. Based on those findings we used the lower amount of peptide for vaccination in Figs. 4+5.

***Query 6:*** *Supplementary Figure 6B: the panel lacks a y-axis label.*

**Response:** The reviewer is correct. We added the information in SFig. 6B.

**REVIEWER #2:**

This reviewer had three major concerns:

***Query 1:*** *The addition of Bl-Eng2 to adjuplex and NP appears to promote enhanced flu-specific T cell responses in terms of numbers for antigen-specific T cells detected in the lung and cytokine production by these cells. Have the authors tested whether this response results in superior protection against challenge with Influenza in terms of reductions in viral titers and survival from infection?*

**Response:** The reviewer raises an important question. We repeated the experiment and measured plaque forming units (pfu) in the lung of Influenza A virus challenged mice. We found that the addition of ΔN rBl-Eng2 to mice vaccinated with NP and adjuplex significantly increased the frequencies and number of NP366-specific CD8^+^ T cells in the total lung, and in the parenchyma (vs. vasculature), and also reduced lung pfu at day 6 post-infection. In addition to augmenting protection, these results show that ΔN rBl-Eng2 exhibits adjuvant properties not only for anti-fungal CD4^+^ T cells but also anti-viral CD8^+^ T cells. We show these new data in Fig. 6 of the revised manuscript and described them in the results and discussion.

***Query 2:*** *Conceptually, one would predict that Bl-Eng2 would be an excellent adjuvant to boost immunity against various clinically relevant fungi but not necessarily other types of pathogens. Influenza infections are certainly of significant clinical importance but focusing the analysis of Bl-Eng2 as adjuvant in this model seems like a random choice and lost opportunity. Their own data shows that Bl-Eng2 basically had no adjuvant activity for the activation of antiviral T cells and NP+Bl-Eng2 failed to activate any T cell responses above background of NP alone (A finding contrary to the stated title suggesting that Eng-2 is an adjuvant for antiviral immunity). Furthermore, without the protection data asked in point 1 we do not know if the statistically significant enhancements in T cell response induced by addition Bl-Eng2 to Adjuplex are biologically significant to antiviral defense. Perhaps Bl-Eng2 is a potent but restricted antifungal adjuvant. Have the authors tested whether truncated Bl-Eng2 can function as an adjuvant to boost superior immunity against fungal pathogens other than Blastomyces?*

**Response:** To accommodate the reviewer’s suggestions, we conducted additional experiments to ascertain whether ΔN rBl-Eng2 boosts vaccine immunity to fungal pathogens other than *Blastomyces.* We chose to test ΔN rBl-Eng2 in vaccination against disseminated infection with *Candida albicans* and against pulmonary *Cryptococcus neoformans* infection. For the *Candida* model we vaccinated mice with Als3 peptide, which has been shown to protect mice against infection when formulated with the Dectin-1 agonist curdlan (J. Immunol. (2012) 188 (11);5636). In the *Candida* model, the addition of ΔN rBl-Eng2 increased the percentage and number of tetramer-positive and cytokine-producing T cells, and reduced kidney CFU at day 4 post-infection and increased survival. In the Cryptococcus model, we vaccinated mice with the protective antigen Cda2. The addition of ΔN rBl-Eng2 reduced lung CFU significantly compared to the control groups. From these data taken together, we concluded that ΔN rBl-Eng2 is an adjuvant that can enhance vaccine induced immunity to multiple fungal pathogens that largely require CD4^+^ T cells for protection and also to a viral pathogen that depends on CD8^+^ T cell mediated immunity.

***Query 3:*** *A lot of the exciting data on Eng2 as novel adjuvant via activation of Dectin-2 have been previously reported in excellent publications by this team of investigators including previous PLoS Pathogens and Mucosal Immunology papers. The novel data presented in the current paper appears to be a narrowing down of adjuvant activity of the Eng2 gene to the C-terminus and need for specific glycosylation signals. Perhaps I have missed other important, new findings in this paper? A further identification of the extent of truncated Eng2 adjuvant activity against a diversity of fungi might help broaden the impact of the current study.*

**Response:** The reviewer is correct. In this paper, we narrow down the structural basis of the Eng2 adjuvant action and Dectin-2 recognition to the O-glycans at the C terminus of the glycoprotein. To accommodate the reviewer’s suggestion on broadening the impact of our discovery, we now also demonstrate that the O-glycans can promote vaccine adjuvant activity against multiple fungal pathogens as well as Influenza A virus.

**REVIEWER #3:**

This reviewer had only minor concerns:

***Query 1:*** *The authors show that transgenic mice expressing CD4+ T cells with TCR specific for protective epitope/shared antigen for Blastomyces dermatitidis, Histoplasma capsulatum, Coccidioides posadasii, Paracoccidioides lutzii, and Paracoccidioides brasiliensis are protected via a Th1/Th17 immune response in the lungs. Do they expect similar outcome with vaccines targeting other fungal infections outside the above-mentioned pathogens (e.g. Cryptococcus or Candida) and known to require similar mechanism of protection?*

**Response:** The short answer is that we now show in new data (Fig. 6) that our adjuvant also augments resistance against both *Cryptococcus* and *Candida.* T helper polarization is principally determined by the cytokine milieu that is present during the priming phase of naïve T cells. The cytokine milieu (signal 2) is largely determined by the pathogen recognition receptors and signaling pathways that are engaged during antigen presentation. For example, we have shown that engagement of Dectin-2 by Bl-Eng2 triggers the production of cytokines that leads to the differentiation of Th1 and Th17 cells (*PlosPathogens* (2017) 13 (8): e1006568). Similarly, in this manuscript when used with different antigens (Calnexin, Bl-Eng2 peptide, Als3 peptide and NP), ΔN rBl-Eng2 induces a mixed Th1 and Th17 cell response by Ag-specific CD4^+^ and CD8^+^ T cells. While optimal host resistance to each fungal pathogen might require a particular T helper profile (*Cryptococcus* and *Histoplasma* immunity depend largely on Th1 immunity, while *Candida* and *Blastomyces* immunity benefit from a mixed Th1 and Th17 cell profile), the mixed Th1/Th17 profile that is elicited by vaccination using antigens with ΔN rBl-Eng2 protects against *Cryptococcus* and *Candida* infection (see the new Fig. 6).

***Query 2:*** *In figure 1, Can the authors comment on the inconsistency in the size of the rBl-Eng2 expressed in P. pastoris in A (between 75-100 kDa) and C (between 100-150 kDa). Also, the E. coli-expressed Bl-Eng2 is not pure. Is it possible the impurities are blocking the activation of the reporter cells?*

**Response:** The reviewer is correct that there does seem to be variation in the way the purified Bl-Eng2 protein from *Pichia pastoris* runs on SDS PAGE. We have not investigated this variation rigorously, but speculate about the reasons. The purified protein may run slightly differently between different gels, and the extent of glycosylation could vary as well. Regarding the possibility that impurities of *E. coli* derived Bl-Eng2 could interfere with the reporter assay, we believe that this is unlikely since recognition and triggering of Dectin-2 reporter activity requires the presence of the carbohydrate recognition domain (CRD). Since E. coli does not glycosylate proteins, any proteins produced by the bacteria should not interfere with the reporter assay. Please also see the response to query #3 by reviewer 1.

***Query 3:*** *On page 16 (in their attempt to investigate if Bl-Eng2 stimulated immunity via C-terminus O-mannans or due to N-terminus CD4+ T cell antigen) and to make it clear, it is best to add a sentence to explain that the adoptively transferred, congenic (CD90.1+) TCR transgenic mice has been vaccinated with Bl-Eng2 combined with each of the tested antigen. Perhaps the sentence can be rephrased as “First, we formulated the immunodominant T cell epitope of calnexin (35) with ∆N rBl-Eng2, vaccinated and then measured T cell expansion and differentiation of adoptively transferred, congenic (CD90.1+) TCR transgenic 1807 cells (SFig. 4).*

**Response:** We regret that this information was unclear. In Fig. 4, we are combining the putative adjuvant (∆N rBl-Eng2) with one or another of two protective antigens (using the corresponding immunogenic peptides) that we have previously identified from *B. dermatitidis*: Calnexin and Bl-Eng2. To read out the development of Calnexin-specific T cells, we use the adoptive transfer system of 1807 T cells. Naïve congenic (Thy1.1+) 1807 cells were transferred into naïve wild type mice prior to vaccination. Mice were then vaccinated with Calnexin peptide and ∆N rBl-Eng2 and the primed T cell response and CFU measured. To measure T cell development following vaccination with Bl-Eng2 peptide, wild type mice were vaccinated with Bl-Eng2 peptide and ∆N rBl-Eng2 and the endogenous Eng2-specific T cells were enumerated by tetramer. No adoptive transfer system was used for the second experimental approach with the Bl-Eng2 antigen. We have modified the text in the manuscript to improve clarity.

***Query 4:*** *Similarly, to enhance clarity and at the bottom of page 16, it would be better to mention reduction in B. dermatitidis lung CFU.*

**Response:** To enhance clarity we indicated that the CFU were fungal lung CFU.

***Query 5:*** *In Fig 4, marking the flow cytometry data panels in C (i.e. lungs, vasculature, and IL 17 and IFN-γ cells (in lungs??)) would also help.*

**Response:** To improve clarity, we added the word “lungs” over the dot plots in Fig. 4C and indicated the vasculature (CD45+) and parenchyma (CD45-) in Fig. 4D.

***Query 6:*** *If Bl-Eng2 and/or the N-terminus truncated protein are proposed as adjuvants, why is it being coupled with IFA? Is this for a depot effect? Have the authors tried Bl-Eng2 without IFA?*

**Response**: The reviewer is correct. We used IFA to create a depot of antigen and/or adjuvant (∆N rBl-Eng2). IFA alone or IFA + peptide failed to induce an Ag-specific T cell response or vaccine induced protection as shown in Figs. 4 and 5 of the manuscript. Thus, we believe IFA alone is immunologically inert and does not provide an adjuvant effect by itself.

For the reviewer’s information, we did vaccinate mice with full length Bl-Eng2 in the absence of IFA or any other adjuvant and did not see evidence of an Ag-specific T cell response by tetramer staining (Fig. 1A below) or cytokine production (Fig. 1B below), and lung CFU was not reduced compared to naïve mice (Fig. 1C below). These data indicate that Bl-Eng2 protein is not immunogenic when delivered without IFA, and is likely degraded rapidly *in vivo*. For these reasons, we chose to formulate the antigens and the ∆N rBl-Eng2 adjuvant in IFA for a depot effect to facilitate immunogenicity.

**Fig 1.** C67Bl/6 mice were subcutaneously vaccinated twice, two weeks apart, with Bl-Eng2 alone, or inside or mixed with GCP particle. Control group was vaccinated with PBS. Two weeks after boost, the mice were challenge with *B. dermatitidis*. At day 4 post challenge, the mice were euthanized, and the presence of Ag-specific T cells (A) and cytokine production (B) were evaluated in the lungs. In addition, the fungal burden (C) in the lungs was evaluated at the same time point. Two-way ANOVA was performed with p < 0.05*.

***Query 7:*** *Is there an explanation why a higher dose of the Bl-Eng2 peptide is less immunogenic and protective against B. dermatitidis?*

**Response:** While this is an interesting scientific question, our main goal in SFig. 5 was to empirically define a dose of peptide that would allow us to evaluate the adjuvant function of ∆N rBl-Eng2. However, it is known that antigen dose and duration can influence the quantity and quality of the ensuing T cell response. For example, a high antigenic load and duration tends to produce highly differentiated effector cells that are not very efficient in transitioning to memory cells. On the other hand, lower amounts of antigen can lead to the formation of less differentiated T cells that have a better chance to survive and turn into memory cells. We did not stain the primed T cells with markers to gain insight into the quality and longevity of the memory T cells.

***Query 8:*** *How long is the vaccine effect expected to last and is a boost dose required or a single dose would result in a protective immune response?*

**Response:** The reviewer raises interesting questions. However, we believe that these are outside the scope of this manuscript. We have not performed a systematic analysis to address durability of T cell immunity induced by this vaccine/adjuvant formulation, the need for boosting, etc.

***Query 9:*** *The data for vaccinating Dectin2-/- mice with Bl-Eng 2 peptide and Degly ∆N rBl-Eng2 is missing from Fig 5. I understand that the expected data would be negative, but would be reassuring to have this extra control for these studies.*

**Response**: We understand the reviewer’s point and agree that it is very unlikely that the outcome of vaccination with Bl-Eng 2 peptide and Degly ∆N rBl-Eng2 in Dectin-2 KO mice would be different than in vaccinated wild-type mice. Since we had limited numbers of Dectin-2 KO mice we were unable to include this additional control group. Nevertheless, we believe that the results in Fig. 5 are strong and convincing even without the requested double negative control group.

In sum, we have modified our manuscript in response to the reviewers’ comments and suggestions. We believe that our new experiments, data, and modifications have greatly strengthened our paper and conclusions.

We hope that the Editors agree that the paper is stronger and is now suitable for publication.

Sincerely yours,

Marcel Wuethrich, PhD
